# After the Trip: A Qualitative Study of Healthy Individuals’ Motor Experiences Following Different Doses of Psilocybin

**DOI:** 10.1002/brb3.71807

**Published:** 2026-10-16

**Authors:** Ruiqi Rachel Luo, David J. Berlowitz, Alexander Bryson, Olivia Carter, Chiranth Bhagavan, Richard Kanaan

**Affiliations:** ^1^ Department of Psychiatry Austin Health University of Melbourne Heidelberg Victoria Australia; ^2^ Department of Physiotherapy University of Melbourne Parkville Victoria Australia; ^3^ Institute For Breathing and Sleep Austin Health Heidelberg Victoria Australia; ^4^ Florey Institute of Neuroscience & Mental Health University of Melbourne Parkville Victoria Australia; ^5^ Department of Neurology Royal Melbourne Hospital Parkville Victoria Australia; ^6^ Melbourne School of Psychological Sciences University of Melbourne Parkville Victoria Australia

**Keywords:** motor function, psilocybin, subjective experiences, thematic analysis

## Abstract

**Introduction:**

Psilocybin is a naturally occurring classic psychedelic associated with widespread changes in brain function and promising therapeutic outcomes in several neuropsychiatric disorders. Complementing these findings, qualitative studies following psilocybin administration have revealed diverse and highly individual effects across multiple domains of subjective experience. However, little is known about its impacts on individuals’ subjective motor experiences. With the tendency of individuals to stay stationary during psilocybin's effects, investigating qualitative motor experiences following low‐to‐moderate doses of psilocybin may offer novel considerations regarding safety and quality of individuals’ psychedelic experiences. We aimed to explore healthy individuals’ subjective experiences after receiving three different doses of psilocybin and conducting motor activity.

**Methods:**

Following the study procedure previously published, semi‐structured interviews were conducted with 12 participants who had completed sessions of motor tasks at different doses of psilocybin, which were then transcribed and thematically analyzed.

**Results:**

Four major themes were developed, each containing sub‐themes: two motor themes, (1) mixed motor messages, including altered motor coordination, control, speed, and reaction time; (2) interacting influences on motor performance, including dose‐dependent effects; and two non‐motor themes, (3) transcendence, altered consciousness, and transformative “mysticism”; and (4) dynamic and enhanced psychological and sensory experiences.

**Conclusion:**

This work contributes to a more comprehensive understanding of psilocybin's multifaceted effects and their relevance to safety, therapeutic potential and future research in motor disorders. The findings will inform a broader dose‐finding study and the design of proposed research on psilocybin‐assisted physiotherapy for refractory motor functional neurological disorder.

**Trial Registration:**

ANZCTR number: ACTRN12621000560897

## Graphical abstract

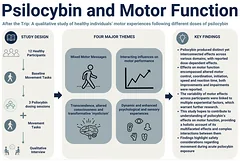

## 1 Introduction

Psilocybin is a naturally occurring alkaloid found in over 100 species of mushrooms worldwide (Passie et al. 2002; De Vos et al. 2021). It belongs to the class of classic psychedelics, which are psychoactive compounds that produce profound mind‐altering effects on biochemical, neural, and psychological levels (van Elk and DB 2022). These broad effects on several domains of brain function have been accompanied by reports of diverse and highly individual subjective experiences. They are thought to contribute to the occurrence of mystical‐type experiences, changes in cognition, affect, and sensory perception (De Vos et al. 2021) and the capacity to increase psychological flexibility (Sloshower et al. 2024). Many qualitative studies to date have offered rich knowledge on how psilocybin helps shape subjective experience in ways that can contribute to personal insights, emotional regulation, and improved therapeutic outcomes of mental health conditions and social or behavioral functioning (Andersen et al. 2021; Breeksema et al. 2020).

Despite substantial attention paid to the phenomenology of psychedelic experiences, to our knowledge, no research has specifically described individuals’ subjective or qualitative experiences of motor function associated with psilocybin use, representing a significant gap in the current literature. Motor function arises from the integration of sensory input and higher‐order cognitive processes that support movement planning, initiation, and feedback. Notably, individuals tend to be still during psychedelic experiences in naturalistic settings (Brouwer et al. 2025), with most clinical psychedelic trials to date instructing participants to relax and lie down during the acute effects (Belser et al. 2017; Griffiths et al. 2016). Given these observations and psilocybin's demonstrated capacity to modulate connectivity across cortical areas, including sensorimotor areas (Madsen et al. 2021), it was postulated that a threshold dose exists beyond which healthy individuals may be unable to complete assigned movement tasks. Therefore, using doses of psilocybin below the typical psychedelic dose range may allow individuals to complete the assigned movement tasks and describe their motor experiences on these doses.

Using reflexive thematic analysis, this study aimed to describe the subjective motor and non‐motor experiences of healthy participants while performing movement tasks after taking different doses of psilocybin. This paper supports the design of a future trial of psilocybin‐assisted physiotherapy for refractory motor functional neurological disorder (FND).

## 2 Materials and Methods

This qualitative study was conducted following the procedure outlined in the previously published protocol, designed to evaluate the safety and feasibility of performing movement tasks during the acute effects of various doses of psilocybin (Bhagavan et al. 2024), with the primary study outcomes reported elsewhere (Bhagavan et al. 2026). Details relevant to the current analysis are provided again here.

### 2.1 Participant Recruitment

Twelve participants were recruited via advertisement in the Austin Health staff newsletter and the Australian New Zealand Clinical Trials Registry (ANZCTR) trial listing (ANZCTR 2025).

Key eligibility criteria included adults aged 18–65 years with no history of FND, and the absence of pre‐existing medical or psychiatric conditions that might exacerbate safety concerns following psilocybin exposure. The full eligibility criteria are provided in the aforementioned protocol (Bhagavan et al. 2024). Ethics approval was obtained from the Austin Health Human Research Ethics Committee HREC/57390/Austin‐2020. All participants provided written, informed consent at the screening visit.

### 2.2 Trial Design

Participants attended three dosing sessions, with psilocybin provided by Usona Institute. Each dose was provided in a randomized order and separated by at least one week. The first six participants recruited received 5, 10, and 15 mg of psilocybin (Block 1), the latter six received 10, 15, and 20 mg (Block 2). A Williams design was used to generate the sequence in which participants were allocated their interventions (Williams 1949). The participants, trial physiotherapists, and statisticians were blinded to the psilocybin dose during the sessions.

Participants completed a series of movement tasks at baseline and during the acute effects of each dosing session at three timepoints (1.5, 3, and 4.5 h post‐dosing). Movement tasks assessed several domains of motor function, including gross motor movements, dexterity, motor speed, reaction time, and interactions with attentional and visuo‐perceptual functions. Mental health support provided included preparation before participants’ first dose, supervision, and psychological integration for each dose. Safety assessments included pre‐dosing vital sign measurements, urine pregnancy, toxicology tests, and medication reviews, as well as vital sign monitoring and adverse event reporting throughout the study.

Semi‐structured qualitative interviews were conducted by study investigators R.K. (*n = *1) and C.B. (*n = *11) one week following the last dosing session. Participants were unblinded to the psilocybin dose for the interview. The interviewer was not blinded. The interviews were conducted either online or in‐person and explored areas of participants’ subjective experience during the study, following an interview guide (Supplementary Information S1). All interviews were audio‐recorded and transcribed verbatim. They were anonymized with potentially identifying information mentioned in the interview and de‐identified with marked generic statements during the transcription process (Braun and Clarke 2013).

### 2.3 Data Analysis

Data were analyzed using reflexive thematic analysis following the approach described by Braun and Clarke (2006). All interview recordings were transcribed verbatim. First drafts were produced by researcher G.O. (*n = *3) and R.L. (*n = *9). Two additional rounds of checking were then carried out by R.L. (*n = *12) to ensure accuracy and consistency, with input from C.B. in areas where the audio was unclear. During this process, data familiarization was achieved through the transcription phase, reading and re‐reading the transcripts. Finalized transcripts were uploaded onto the qualitative data analysis software NVIVO (release 15.1.0) to generate first‐level codes (NVivo 15 2023). Coding followed an inductive approach, working with the data from bottom up. Initially, two transcripts were selected at random and independently coded by C.B. and R.L. in a randomized order. The dually coded transcripts were then collaboratively reviewed by C.B. and R.L. as a form of sense‐checking. After this, R.L. completed the initial coding frameworks for all the transcripts through an iterative process. Codes were collated to generate initial themes and subthemes primarily on a semantic level. The themes were then reviewed and defined so that they could provide a narrative that effectively encapsulated participants’ experiences.

## 3 Results

Twelve participants received psilocybin and completed the qualitative interview (seven males, five females, aged 22–39 years), and the interviews lasted from 12 to 25 min. One of these participants completed two dosing sessions before withdrawing from the study, but completed the interview. The remaining eleven participants completed all three dosing sessions. Five participants had a biomedical background and one was deaf and was assisted by an interpreter during the interview.

Thematic analysis of the transcripts yielded four overarching themes: 1) mixed motor messages; 2) interacting influences on motor performance; 3) transcendence, altered consciousness, and transformative mysticism; and 4) dynamic and enhanced psychological and sensory experiences. Although this paper aims to focus on motor elements of psilocybin experiences, the non‐motor themes three and four are included as individuals’ psychological, physiological, and sensory experiences are closely connected to their motor functioning. Each major theme was further divided into sub‐themes as detailed below. Excerpts of transcripts are provided to demonstrate these themes from the participants’ perspectives.

### 3.1 Mixed Motor Messages

Impacts on motor function were extensively discussed by all participants, with references to motor domains involving control, coordination, initiation, movement speed, and reaction time.

- “My limbs weren't doing what I thought they were doing”

Five participants remarked on the impairment of motor control during the acute effects of psilocybin, which was commonly thought to result from attentional deficits. At times, these deficits were ameliorated when participants consciously directed their attention toward the motor tasks. Interestingly, one participant attributed reduced motor control to altered sensory perception.

PSY_HC_01: *“…it just felt slightly clumsy, almost as if my hands were covered in oil and they would slide… they weren't doing what I wanted them to do or what I thought they should do… a bit sort of, the disconnection between… what I thought my arm should feel like and what it felt like and also how it moved compared to what I thought it… should be like.”*

However, several participants reported gaining control once they had overcome the initiation phase of movements. Five participants in particular noted increased effort or delay in motor initiation.

PSY_HC_10: *“…once… I've gotten pass initiation phase… then [motor control] was easier, it was sort of the momentum going.”*

All participants commented on their motor coordination. There was an approximately even distribution between individuals who experienced improvements, usually evidenced by enhanced balance, and those who reported a decline in motor coordination. Four participants observed improvements.

PSY_HC_03: *“…some days where I was able to do the tandem stance… and I couldn't do that at my baseline measure. And I wonder if that came about from the fact that I was able to sort of internalize and focus on the movements and the postural corrections…”*

Motor coordination, specifically balance, was compromised in three participants.

PSY_HC_10: *“…the balance [task was] more challenging.”*

b. “It would just take longer”

Nine participants discussed changes in the speed of their movements during the sessions, with the majority reporting a slowing of motor activities. In some cases, this was partly due to changes in cognitive function such as reduced concentration level or diminished motivation.

PSY_HC_09: *“I didn't remember that I had to be going at my maximum speed, so I'd kinda just be… throwing the bricks thinking about other things… being a lot slower with throwing the bricks.”*

However, in one case, the participant observed slowed movements despite having increased cognitive speed:

PSY_HC_04: *“…my movements were slow, but somehow my mind was able to comprehend what symbol to put next to the number… I had no idea… how my mind was working so fast…”*

c. “I was reacting a bit slower”

Seven participants shared that their reaction time changed compared to baseline; approximately half of these participants reported being able to catch the ruler during the reaction time drop ruler test (Bhagavan et al. 2024) or noticing improvements in their reaction time.

PSY_HC_13: *“the reaction time one… on the twenty‐milligram dose there was a time when I almost felt slightly masterful… I was… doing almost slightly quicker than on average.”*

Several participants mentioned that this improvement or the lack of change in reaction time occurred because they were able to dedicate their attention to the task, as the task had a shorter duration compared to the others.

PSY_HC_12: *“That I only really had to focus for a very short period of time, I could divide my undivided… attention, like closing my fingers on the ruler, and I did perform the best… with the highest dose”*

On the other hand, some participants observed reductions in their reaction time.

PSY_HC_03: *“I think my… general reaction… when yourself or the physiotherapist walked into the room, I was a bit slower… normally I'd probably be up and straight away looking, whereas I was reacting a bit slower…”*

### 3.2 Interacting Influences on Motor Performance

Participants often contextualized motor changes in relation to cognitive function and other influencing factors. Most of them identified a dose‐dependent relationship as well.

- “Your mind [is] elsewhere”

Concentration was identified as a key factor in motor performance. Many experienced increased distractibility as a result of being mentally occupied by other concurrent experiences. This led to impaired motor functioning, such as reduced movement speed. As such, repeated instructions were reported as beneficial in redirecting attention and improving task engagement.

PSY_HC_12: *“I could also get very distracted and, one thing would lead to another in my brain… you'd find that you slow down the movement… because you thinking about the things to the point where you can almost stop doing the task…”*

On the other hand, two participants exhibited a state of hyperfocus, whereby their attention was directed to normally automatic movements. This heightened awareness brought typically subconscious processes into conscious focus, which appeared to enhance motor functions.

PSY_HC_03: *“… I sort of entered this state where I was hyper‐focused on the movements… to correct my balance during those performance tasks… I know certainly were not under the influence, I wouldn't be thinking about so much the movements… and I think that helped me a little bit in my balance.”*

b. “Wrestling with… carefree attitude”

Reduced concentration was suggested to have influenced participants’ motivation level during the movement tasks, which in itself was a critical determinant of participants’ performance as well.

PSY_HC_09: *“… because what's going on in my head was just so… crazy, I was way more absorbed in that, than in the tasks I was doing… I couldn't bring myself to care, and… to really try as hard as I could, which I can when I am like sober.”*

Decreased motivation was also attributed to other factors, including the repetitive nature of the tasks, carelessness, or negative attitudes towards the tasks.

PSY_HC_03: *“…the importance of the task was less… for some tasks you should be trying to complete them as quickly as you could. And that was sort of wrestling with this… careless, or carefree attitude… so that probably impacted on the performance…”*

However, two participants were able to mentally re‐steer themselves during the session. They reported that psilocybin enabled them to gain a sense of love, from which they could rediscover a sense of motivation and appreciation for the tasks.

PSY_HC_06: *“…I was feeling immense love… and that's when I felt like I really wanted to do this, I wanted to do better than I did last two times.”*

c. Sensory, physiological, and environmental contributors

Other reported experiences were also identified as contributing factors to participants’ motor changes. Links were drawn between sensory effects of psilocybin and participants’ performance in motor tasks:

PSY_HC_01: *“When I got off the bed and started walking, they felt a bit sort of springy in my legs, and when I did the walking task, they felt funny.”*

Others alluded to concurrent physiological experiences when describing their motor performance.

PSY_HC_12: *“…I felt… uncomfortable I didn't know if I wanted to rest or move around… I was too tired almost (chuckles) to pick it up, and my fingers were very sweaty and… it just wasn't working for me…”*

Set and setting also played a role in participants’ subjective motor experiences, expectations, and mindset prior to the movement tasks shaped participants’ impressions of their motor performance:

PSY_HC_03: *“…my motor control was not as great, but… I was expecting that to happen… because I sort of came in anticipating [motor control to be] potentially affected in that space… it met my expectations, so to me it probably didn't feel as severe.”*

d. “I can't feel where [things] are”

Space distortion as an element of ‘mystical’ experience was commonly reported to have affected participants’ motor performances (Griffiths et al. 2006). Descriptions of this included impaired spatial awareness and proprioception.

PSYC_HC_01: *“…something just didn't quite feel right in my hand, it feels too big or…”*

These perceptual changes in turn affected participants’ performance in the subsequent movement tasks, requiring them to engage senses that were not normally needed, such as tactile feedback, to complete the tasks.

PSY_HC_03: *“I found it really hard to judge depth and where the partition was. And the only way I was able to complete that task… was to… rest my wrist on the partition as a tactile feedback.”*

e. “Twenty milligrams…felt more tricky”

Eight participants noticed dose‐dependent effects on motor function, with most participants describing increasing difficulty with movement tasks with higher doses of psilocybin.

PSY_HC_10: *“Definitely a dose‐response effect with the higher dose being… more challenging to perform the task.”*

However, this trend was not universal across all participants. For example, one participant explained that she had more motor control after receiving 10 mg of psilocybin compared to 5 mg, possibly due to developing tolerance or habituation to the substance. Another participant identified mindset as a contributing factor to motor decline, causing poorer performance during 10 mg compared to 15 mg.

PSY_HC_13: *“…the second physiotherapy session [with 10 mg of psilocybin] … was more difficult because… I felt like a lot happened in the interim period.”*

Table 1 shows a summary of dose‐related changes in motor functioning. However, it should be noted that some accounts of changes in motor function were non‐specific, as the semi‐structured nature of the interviews did not consistently elicit direct comparisons between individual dose levels.

**TABLE 1:** Summary of dose‐related changes in motor functioning.

Motor sub‐theme/dose	5mg	10mg	15mg	20mg	Dose‐unspecified^a^	Summary of dose‐related changes
Overall motor functioning	No change (*n = *3)	Reduced (*n = *3); no change (*n = *1)	Reduced (*n = *5); no change (*n = *1)	Reduced (*n = *4)	—	Increasing impairment with higher doses
Balance	—	Reduced (*n = *1)	Reduced (*n = *2)	Reduced (*n = *1)	Improvement (*n = *4); impairment (*n = *3)	Possible increase in impairment with dose, but limited reports
Fine motor function	Reduced (*n = *1)	Reduced (*n = *1)	Reduced (*n = *1)	No change (*n = *1)	Impairment (*n = *3)	Inconsistent; no clear dose‐dependent response
Motor control	Reduced (*n = *1)	Reduced (*n = *1); no change (*n = *1)	Reduced (*n = *2)	No change (*n = *1)	Improvement/ no change (*n = *3); impairment (*n = *5)	Inconsistent; no clear dose‐dependent response
Movement speed	—	Reduced (*n = *1)	Improved (*n = *1)	—	Improvement/ no change (*n = *2); impairment (*n = *9)	Inconsistent; no clear dose‐dependent response
Reaction time	Reduced (*n = *1)	Reduced (*n = *1); no change (*n = *1)	Reduced (*n = *2)	No change (*n = *1)	Improvement (*n = *4); Impairment (*n = *3)	Some evidence of impairment at higher doses, but inconsistent

*Note*: Counts represent participants who explicitly described a change at the specified dose level. A blank cell indicates that no explicit dose‐related changes were mentioned, rather than an absence of effect.

^a^Indicates participants who reported subjective improvements or impairments but did not specify the dose associated with these effects.

### 3.3 Transcendence, Altered Consciousness, and Transformative ‘Mysticism’

Accounts of ‘mystical’ experiences included ego‐dissolution, death‐like experiences, sense of love, and ineffability.

- “I am not actually this body, I am a soul”

Several participants reported experiencing ego‐dissolution following acute administration of psilocybin. Descriptions of such experience were strongly tied to a perceived dissociation or detachment of the sense of self from the body. In this experience, participants felt that their “feeling of embodiment” had changed, where the ego had disintegrated from the body, becoming a separate entity. Furthermore, this sometimes gave rise to a sense of union with their surroundings.

PSY_HC_11: “*I felt like… my sense of self, was almost gone…you become… lost… in… the… sensation that you were feeling… I was like kind of, transformed, in like… the world of swirls …what I was feeling was like taking over … in a way that… I was just, becoming one with them*.”

One participant felt that the body became a limitation that impeded their access to a higher realm.

PSY_HC_12: “*My physical body was… a constraint… holding me back, and I couldn't truly let go… I couldn't reach that higher plane because my body was there… I wanted… to let go, of… my body*.”

b. Death, Rebirth, and Different Lives

Two participants reported experiencing death‐like experiences, though the nature of these experiences varied. This was perceived both physiologically and sensorily. Both described physical sensations, with one participant recounting the feeling of their “heart [having] stopped”. One participant also recounted visual imagery associated with the process of dying. However, for both participants, these experiences did not elicit any significant negative emotions such as anxiety, distress, or negative thoughts.

PSY_HC_04: “*There were certain things that I felt, like my heart stopped beating… and the liquid coming out of my mouth, I felt that one rather than seeing anything. But the other one… was kind of a blurry vision… I saw myself dying, and someone was trying to resuscitate me*.”

One participant recalled a sense of being reborn into different bodies, inhabiting different bodies and living multiple lives. In the sessions, they witnessed different versions of themselves co‐existing, further contributing to their sense of time distortion as mentioned above.

PSY_HC_04: “*I felt living different lives… and different realities also as different persons…. I saw my younger self maybe about 3 or 4 years old… and then there was another version of myself… 20, 21… I saw them standing side by side, doing their own thing… I know both of them are part of myself… they're both existing simultaneously together… if I look into now, it is not possible [given how time works]*”

c. “Time… collapsed”

Time distortion emerged as a common theme across participants. Among those who experienced time distortion, most indicated that sessions in which they received higher doses “felt longer”. One participant described the “collapse” of time after witnessing multiple versions of herself at different ages coexisting in a single point in time—an experience that she “wasn't able to comprehend.”

PSY_HC_04: “…*that person [who] was three or four years old needed to exist for me to be here… because if she wasn't there I wouldn't have been here… So…it all distorted… how the time really was… the way I perceive how [time] works is past, present, and future… it really doesn't work like that*.”

In addition, most participants identified a dose‐dependent relationship, whereby the time distortions were more pronounced with increasing doses of psilocybin administered.

d. “I was feeling immense love”

Four participants noted experiencing feelings of love during the dosing sessions. This was articulated in various ways and directed towards different entities depending on the individual.

Most participants classified their feelings as “love” or “immense love”. One participant referred to it through more nuanced terms—“benevolent presence”, where there is a combination of a sense of comfort, care, and quasi‐spiritual presence, which can be broadly understood as an experience of human connection and love.

PSY_HC_13: “*I had a real presence…like a sense of something was next to me… people in the abstract sense… were looking out for me… in a very non‐physical way… that was really nice… it… almost felt like, somewhere between abstract and physical because it really felt like there was something there… that was just a very benevolent… presence… I think it's just human love that I was feeling*.”

Nature was also a salient notion associated with love. Participants reported thoughts and visual imagery involving natural elements, such as “thoughts of waves and undulating mountain tops”. These experiences were frequently described as “awe‐inspiring” and fostered a sense of “connectedness to [the] natural world”.

e. “An experience that I cannot describe”

Over half of the participants noted a sense of ineffability, in which they were unable to articulate their experiences or find words to adequately convey them. Some participants also acknowledged struggles in unpacking their emotions and making sense of the experience.

PSY_HC_09: “*[the experience is] not something that i can put into words particularly eloquently*.”

### 3.4 Dynamic and Enhanced Psychological and Sensory Experiences

Participants reported diverse and heightened effects on emotional and sensory domains following psilocybin administration. Psychological benefits obtained from these “journeys” were often perceived as personally meaningful and influential in their lives. Narratives of heightened sensory experiences mainly consisted of changes in vision, hearing, and taste. In addition, most participants also remarked on undesirable physiological effects induced by psilocybin.

- “Moments of positivity and negativity”

A range of emotions was elicited during the acute effects of psilocybin, which can be broadly categorized as either positive or negative.

Common positive descriptions included feeling “euphoric”, “enlightened,” and “safe”. Of these descriptions, a “sense of calm” was most frequently expressed (*n = *5), which was often associated with a sense of relaxation and inner peace. This sense of serenity and inner peace was often accompanied by an experience of pure awareness, characterized by the perception that there is “just [their] consciousness and everything”. This phenomenon arguably reflected a sense of internal unity, which may also be considered an aspect of ‘mystical’ experiences (Griffiths et al. 2006).

PSY_HC_04: “*I felt like my whole body was calm, there's nothing else… I would have kept on sitting in the room for…more than 5 hours [and] not [move] at all. I felt like there wasn't anybody, just my consciousness and feeling everything. There's nothing outside of me*…”

Despite many accounts of positive emotional experiences, negative emotions were noted as well, including feelings of dysphoria and anxiety. Four participants indicated feeling emotionally overwhelmed and disoriented during the sessions, often attributed to a dramatic increase in the volume and speed of their thoughts. For instance, one participant used an analogy of being on a rocket:

PSY_HC_14: “*I felt like I was on, a rocket… the wind was just rushing by and you don't know where to look because everything is just travelling so fast and [I] feel like I don't have, time to look… [things were] whooshing straight through and it's very quick… it seemed overwhelming*.”

All participants experienced multiple types of emotions throughout the sessions. Most notably, two participants reported oscillating emotional states, cycling between brief moments of sadness and happiness.

Moreover, the set and setting, especially music, appeared to play an important role in shaping participants’ psychological experiences, where, for example, high‐frequency music would lead to a different emotion. One participant also commented on the ability of music to give them directions during the session, which helped him to shift away from negative emotions.

PSY_HC_14: “…*ambient music… helped me to be able to re‐steer… I felt that I had directions… if it wasn't for that music or any music at all… I could've spun out of control… gone off track and possibly panic… so I am quite glad that I had the music there*.”

b. “I got a lot out of it”

Most participants (*n = *9) revealed that the dosing sessions offered them psychological benefits, with some of these gains persisting beyond the course of psilocybin. These can be categorized into 1) newfound perspectives, 2) clarity of mind, and 3) changes in interpersonal relationships.

Seven participants indicated that psilocybin allowed them to gain new perspectives, mainly around their approach to life, and facilitated greater self‐insight.

PSY_HC_04: “…*[this experience] just opened up a whole new [case about] what I think, how we come into the world, how we die and how we live our life it completely, I'd say [it] changed my whole perspective*.”

Three participants reported that psilocybin provided an opportunity for them to let go of material concerns and everyday worries. This was often associated with the occurrence of ‘mystical’ phenomena during the sessions. For one individual, this shift was linked to a greater appreciation for natural beauty following an experience of ego‐dissolution and visual imagery of an Ecuadorian cave:

PSY_HC_12: “*If there's such things that exist, so awe‐inspiring, and beautiful and magnificent as these caves in Ecuador that would have such natural beauty… I shouldn't be worried about these… small insignificant things in my life that aren't gonna worry me in five months’ time*…”

Clarity of mind appeared to mostly stem from the sense of calm and serenity participants gained throughout the dosing sessions. During this state, participants often described finding the world and “things being simpler”, where they felt devoid of responsibilities.

PSY_HC_03: “*My mind… was quite clear during the peak… I didn't have all of the thoughts… responsibilities and things that I would normally have in a day crowding my mind. I knew they existed, but I just wasn't ever thinking about them*.”

Of these participants, many observed that they were able to take these benefits beyond the course of psilocybin and integrate them into their everyday lives. While most participants discussed these in general terms, a few provided specific examples to illustrate their experiences.

PSY_HC_03: “…*a situation [arose] in my work where I was going to need to have a difficult discussion… and I spent some time in that first session thinking about how to have that conversation… I think that really helped because… [for] those situations [that] I find quite difficult normally… I was able to come up with some thoughts that I then built upon the next day before I went on to have that conversation*…”

Furthermore, seven participants reflected on the perspectives psilocybin brought to them in terms of interpersonal relationships. This fostered greater appreciation, connection, and compassion for others.

PSY_HC_03: “…*there were moments during the first and second session where I reflected on relationships and was appreciative of the relationships I had… [it was] an opportunity to remind myself and not take those relationships for granted*.”

One participant was able to integrate the perspectives into his life, which enabled him to improve the quality of his relationships with friends.

PSY_HC_14: “…*not only is it those changes in yourself but you [are] noticing there [are] changes in the dynamics with your relationships as well around you*.”

c. “The visuals were so strong”

All participants reported experiencing altered visual phenomena during the acute effects of psilocybin. Across these accounts, seven participants recalled having visual imagery. This ranged from geometrical shapes to landscapes to vivid scenes, which often occurred with eyes closed. While this experience was generally understood by participants as a result of their visual sensation, one individual noted difficulty distinguishing them from imagination.

PSY_HC_12: “*when I closed my eyes… the surface the roof the walls were made out of this cushion‐y… mushy brain material … and in the very centre of that setting was…barber chairs… and there was a man, sitting down there… a male, non‐descript though, there was no discernible facial features, or expressions or mood or vibe or about it… I could just see them swimming around in the chair… putting his hand up… pointing and touching like there was an array of iron man futuristic see‐through tablets just in front of him… almost like he was… choosing memories to recount… that was probably the most… absurd or alien‐like… vision… throughout the experience*…”

Enhanced visual experiences were endorsed by all participants, often occurring with eyes open. “Things were more vivid and colorful,” and colors also became “more saturated, more bright”—an experience that many participants enjoyed.

PSY_HC_12: “*I started to… have. some visuals… the way I described it was watching a three‐D movie without the glasses on, and you can see the red and the blue lines trailing after all the… moving images*…”

Four participants noticed a dose‐dependent effect. With higher doses, visual effects had increased intensity. In one case, the speed at which imagery formed increased as well.

PSY_HC_08: “…*amongst the three sessions… the [intensities] of the patterns are definitely different, [on 15 mg it] is very optimal… [on 20 mg] the pattern was very strong so it came very quickly and [disappeared] very quickly*…”

d. “Enhancement of sounds” and more

Most participants discussed enhancements in other sensory modalities. This included auditory sensitivity (*n = *5), where participants remarked on how they “could hear things more”. Dissimilar to hallucinatory experiences, participants explained that they became more aware and could hear ambient sounds more clearly, whereas these noises would have normally been imperceptible in everyday contexts.

PSY_HC_06: “*I could hear [people] talking or laughing… even the trip of water from… the air conditioner, I could hear everything but it felt like it was coming from… nearby… that's the kinds of the sounds [that are] so faint that I would not even know that they were there, but I could hear them during the dose*.”

Five participants also mentioned heightened tactile and gustatory sensations.

PSY_HC_03: “*When I did have those jelly beans, I really appreciated it… I could really taste them… they tasted different to. regular jelly beans*…”

Several participants experienced synesthesia as well, where sensory modalities such as hearing, vision, and touch crossed over, especially while they were listening to music.

PSY_HC_10: “*Sound had [an]… added a dimension to it… [like] listening to music in like four‐D or five‐D and… so it kinda felt like there was a sense of feeling and seeing the music… on… top of listening as well*.”

e. “Wasn't feeling too well”

Although many participants endorsed pleasant sensory experiences, almost all participants identified undesired physiological effects as well. The most common effects were fatigue and altered muscle sensations. Most participants reported fatigue as a non‐specific effect of psilocybin, while others saw it as a consequence of the emotionally and physically intense nature of the experience. In several cases, this negatively impacted participants’ engagement with the movement tasks.

PSY_HC_10: “…*all I wanted to do first time around was sleep…… it wasn't because the balancing was difficult it was more that I was just too sleepy, and too tired, don't want to do anything*.”

Several participants talked about altered muscle sensations, which included pain, stiffness, and restlessness in the legs. One participant shared that there was an unusual sensation in their legs, which made them feel “funny” and “springy”. These altered sensations also influenced performance during movement‐based activities.

PSY_HC_03: “*I found my legs get really stiff, particularly my abductors on the outside… they were getting a bit achy and tight from such a short activity… each time… I would just shake my legs out, ‘cause they were feeling quite stiff and sore. And that instantly wasn't a problem once I'd done that*…”

Other common physiological effects mentioned were nausea, headache, and reduced appetite. Notably, four participants compared the psilocybin experience to being “drunk”, referencing both physical and cognitive effects, such as “disinhibition” and slowed mental processing.

PSY_HC_08: “…*The state of description would be, that you got really really drunk*.”

## 4 Discussion

This study explored the motor and non‐motor experiences of healthy individuals participating in a double‐blind, randomized trial of the effects of three different doses on motor task performance. In the study, individuals described a range of experiences that could be broadly classified into four themes spanning altered movements, factors that affected motor functions, ‘mystical’ phenomena, and complex yet meaningful psychological and sensory experiences. To our knowledge, this is the first work describing qualitative experiences of motor functions in the context of psilocybin use.

Across participants’ accounts, particular emphasis was placed on motor control, coordination, initiation, speed, and reaction time. Variable impacts were observed, including both impairments and improvements. This was primarily attributed to cognitive changes, specifically diminished attentional capacity—a phenomenon observed in other studies as well (Turton et al. 2014). In the context of motor experience, this attentional deficit appeared to stem from concurrent experiences that diverted much of participants’ attentional resources. In turn, being more absorbed in concurrent experiences reduced their motivation to engage or perform well in their assigned movement tasks. This suggests that the immobility seen in psychedelic experiences (Brouwer et al. 2025; Belser et al. 2017; Griffiths et al. 2016) may be affected by impairments in primary motor function as well as via the altered cognitive state and motivational level that deprioritized movement during the course of the drug effects. Furthermore, the influence of non‐motor experiences such as ‘mystical’ experiences, physiological, and sensory changes is evident across participants’ accounts of motor experiences. These narratives highlight the complex integration of sensory and environmental inputs involved in the conceptualization, planning, and execution of movement. This provides further insight into how motor actions may be shaped by psilocybin's indirect effects on broader psychological and physiological processes.

In addition, dose‐dependent effects were reported for most motor domains, though this was not universal across all participants. Along with the inconsistent pattern of performance, this indicates that multiple contextual and personal factors, such as familiarity effect, drug tolerance, and concurrent psychological and sensory experiences, may have influenced these outcomes. Variability in performance may also be attributed to the order of dosing sessions, for example, to increasing familiarity with the tasks and psilocybin experience, or decreasing fatigue with repeated doses. The relative contribution of these factors to the observed findings remains unclear, warranting the need for future research using controlled settings and clearly defined variables to more precisely quantify psilocybin's effects on motor function.

Psychedelic research has been traditionally conducted with participants in stationary positions, thereby excluding motor experiences from the broader picture. Consequently, little data exist on the safety and feasibility of performing motor tasks under the influence of psilocybin. In this study, doses below the typical psychedelic treatment range were used to engage participants in movement tasks and explore the qualitative effects of psilocybin on motor experience at these doses. This hopes to contribute to the current understanding of psilocybin's effects on human motor function, while providing a holistic account of its multifaceted effects on brain function, potential psychological and therapeutic mechanisms, and the complex interactions between them. The present findings speak to the potential motor difficulties but also the capacity to perform motor activities during the course of psilocybin, highlighting how these changes relate to other aspects of participants’ subjective experiences.

Mystical experiences are among the most unique experiences induced by psychedelics. The findings of this study further supported the current literature but offered a novel extension—mystical experiences also appeared to have influenced participants’ engagement in the movement tasks, with impacts varying depending on the specific nature of the experience. For instance, space distortion prompted individuals to rely more on alternative sensory input to complete the tasks, while feelings of love and unity fostered newfound appreciation and motivation for the movement tasks.

Consistent with existing literature, participants frequently alluded to a range of physiological effects such as fatigue, headache, and muscle sensations (Griffiths et al. 2016; Yerubandi et al. 2024). In the context of motor experiences, these undesired physiological effects made the movement tasks more difficult, thereby impacting participants’ overall motor performance. Changes in sensory perception were also indirectly tied to participants’ motor experiences, for example, by occupying attentional faculties, contributing to the increased distractibility and corresponding motor impairments. Future research may need to consider accommodating the influence of these somatosensory experiences if motor function is a key area of interest.

These insights raise important considerations for the safety and feasibility of engaging in motor activities following low‐to‐moderate doses of psilocybin, both in naturalistic settings and future clinical trials. Taken together, they could inform future research investigating its therapeutic potential in conditions with motor dysfunction, such as FND (Bhagavan et al. 2024; Bryson et al. 2017), cerebrovascular disease, and acquired brain injury (Allen et al. 2024; Nardou et al. 2023). In particular, these findings have direct implications for the design of a planned subsequent study of psilocybin‐assisted physiotherapy for individuals with refractory motor FND. FND‐specific physiotherapy recruits higher‐order cognitive processes, including attentional redirection, engagement with psychoeducation, and strategies to facilitate automatic movement. Our findings of dose‐dependent changes in attention and sensory perception, fatigue, and mystical‐type experiences that interfered with movement performance suggest that a lower‐than‐conventional therapeutic dose of 25 mg may better preserve the cognitive capacity required for meaningful physiotherapy. These findings complement our quantitative outcomes, which observed challenges with feasibility and impairments in motor function in tasks combining multiple motor and/or cognitive functions at 20 mg (Bhagavan et al. 2026). These findings informed the selection of a 15 mg dose for a treatment arm that incorporated physiotherapy during the acute drug effects for the subsequent FND study, and identified motor function domains that warrant further assessment during this treatment approach, such as movement initiation, balance, and control (Bhagavan et al. 2025).

### 4.1 Strengths and Limitations

This study has several limitations. This sample was self‐selected, with nearly half of the participants having a biomedical background. This is likely to limit generalizability, as these individuals may have particular perspectives or experiences that are not representative of the broader population. There was at least one week's interval between the last dosing session and the interview, so fading memory and recall bias may have affected recollection of participants’ experiences. Post‐event processing may have further shaped participants’ retrospective accounts and affected the accuracy of self‐reported responses during interviews. Power imbalances are likely, given interviewers’ roles as consultant psychiatrists, which potentially skewed responses towards perceived desirable narratives, particularly in those from a biomedical background, and influenced R.L.’s interpretation as a medical student. In addition, being unblinded, the interviewers’ knowledge of the study aims may have inadvertently influenced the direction of the interview and guided participants towards anticipated findings. To address this, reflexivity was applied and the semi‐structured interview guide was carefully developed and consistently followed.

Despite these limitations, the flexibility of reflexive thematic analysis enabled theme development from a rich dataset and the conceptualization of varied experiences in an understudied area of research (Braun and Clarke 2006). This approach was chosen to align with the research goal of exploring subjective experiences of the acute effects of psilocybin, making it valuable for an understudied topic. Taken together, these will help to identify emerging areas of research that warrant further exploration.

## 5 Conclusion

This qualitative study provided an overview of psilocybin's effects on individuals’ subjective motor experiences, encompassing both direct influences, such as motor control, coordination, initiation, speed, and reaction time, as well as indirect effects through changes in cognition and sensory experiences.

## Author Contributions

**Ruiqi Rachel Luo**: writing – review and editing, writing – original draft, software, formal analysis, methodology. **David J. Berlowitz**: writing – review and editing, conceptualization, funding acquisition. **Alexander Bryson**: writing – review and editing, conceptualization, funding acquisition. **Olivia Carter**: writing – review and editing, conceptualization, funding acquisition. **Chiranth Bhagavan**: writing – review and editing, project administration, data curation, supervision, validation, investigation. **Richard Kanaan**: data curation, supervision, project administration, writing – review and editing, conceptualization, funding acquisition, investigation. All the authors read and approved the final manuscript.

## Funding

This project received funding from the Medical Research Future Fund, APP2012410.

## Ethics Statement

Ethical Approval Was Awarded by the Austin Health Human Research Ethics Committee HREC/57390/Austin‐2020. The authors assert that all procedures contributing to this work comply with the ethical standards of the relevant national and institutional committees on human experimentation and with the Helsinki Declaration of 1975, as revised in 2008.

## Consent

Participants were invited to a screening visit with the study physician, during which informed consent was undertaken. Information regarding study procedures, possible benefits and risks was presented in a neutral and balanced manner. Informed consent was obtained for the permission to use de‐identified data for this study and secondary uses, including informing future related projects and sharing safety data with the Usona Institute. Individuals willing to proceed with the study will provide a dated signature on the PICF.

## Conflicts of Interest

Richard Kanaan is a member of the scientific advisory board of Psychae, a non‐profit psychedelic research institute. Chiranth Bhagavan has received funding from the Graham Burrows Travelling Scholarship, University of Melbourne and has received grant funding from the RANZCP Foundation, the Royal Australian and New Zealand College of Psychiatrists. Olivia Carter has received funding from the Perception Restoration Foundation. Richard Kanaan, Olivia Carter, David J. Berlowitz, and Alexander Bryson received funding from the Medical Research Future Fund for research into psychedelics in functional neurological disorder, MRF2012410. The remaining authors declare no conflicts of interest.

## Supporting information




**Supporting File 1**: brb371807‐sup‐0001‐SuppMat.docx

**Supporting File 2**: brb371807‐sup‐0002‐SuppMat.docx

**Supporting File 3**: brb371807‐sup‐0003‐SuppMat.docx

## Data Availability

De‐identified safety data will be shared with the Usona Institute, while access to the full final trial dataset will be restricted to study investigators and other relevant regulatory bodies. Intellectual property generated from this study will be owned by the University of Melbourne.
