# Supplementary material for: After the Trip: A Qualitative Study of Healthy Individuals’ Motor Experiences Following Different Doses of Psilocybin
Source: Brain Behav. 2026 Oct 16;16(10):e71807. doi: 10.1002/brb3.71807 (PMC13674734; doi:10.1002/brb3.71807)
Supplement: Supplementary file 1 [file BRB3-16-e71807-s001.docx]

**Supplementary material**

**1. Transcendence, altered consciousness and transformative ‘mysticism’**

Accounts of ‘mystical’ experiences included ego-dissolution, death-like experiences, sense of love and ineffability.

a. “I am not actually this body, I am a soul”

Several participants reported experiencing ego-dissolution following acute administration of psilocybin. Descriptions of such experience were strongly tied to a perceived dissociation or detachment of the sense of self from the body. In this experience, participants felt that their “feeling of embodiment” had changed, where the ego had disintegrated from the body, becoming a separate entity. Furthermore, this sometimes gave rise to a sense of union with their surroundings.

PSY_HC_11: “*I felt like… my sense of self, was almost gone…you become… lost… in… the… sensation that you were feeling… I was like kind of, transformed, in like… the world of swirls …what I was feeling was like taking over … in a way that… I was just, becoming one with them.”*

One participant felt that the body became a limitation that impeded their access to a higher realm.

PSY_HC_12: “*My physical body was… a constraint… holding me back, and I couldn’t truly let go… I couldn’t reach that higher plane because my body was there… I wanted… to let go, of… my body.”*

b. Death, Rebirth and Different Lives

Two participants reported experiencing death-like experiences, though the nature of these experiences varied. This was perceived both physiologically and sensorily. Both described physical sensations, with one participant recounting the feeling of their “heart [having] stopped”. One participant also recounted visual imagery associated with the process of dying. However, for both participants these experiences did not elicit any significant negative emotions such as anxiety, distress, or negative thoughts.

PSY_HC_04: “*There were certain things that I felt, like my heart stopped beating… and the liquid coming out of my mouth, I felt that one rather than seeing anything. But the other one… was kind of a blurry vision… I saw myself dying, and someone was trying to resuscitate me.”*

One participant recalled a sense of being reborn into different bodies, inhabiting different bodies and living multiple lives. In the sessions, they witnessed different versions of themselves co-existing, further contributing to their sense of time distortion as mentioned above.

PSY_HC_04: *“I felt living different lives… and different realities also as different persons…. I saw my younger self maybe about 3 or 4 years old… and then there was another version of myself… 20, 21… I saw them standing side by side, doing their own thing… I know both of them are part of myself… they're both existing simultaneously together… if I look into now, it is not possible [given how time works]”*

b. “Time… collapsed”

Time distortion emerged as a common theme across participants. Among those who experienced time distortion, most indicated that sessions in which they received higher doses “felt longer”. One participant described the "collapse" of time after witnessing multiple versions of herself at different ages coexisting in a single point in time – an experience that she “wasn’t able to comprehend.”

PSY_HC_04: “*…that person [who] was three or four years old needed to exist for me to be here… because if she wasn't there I wouldn't have been here… So…it all distorted… how the time really was… the way I perceive how [time] works is past, present, and future… it really doesn't work like that.”*

Additionally, most participants identified a dose-dependent relationship, whereby the time distortions were more pronounced with increasing doses of psilocybin administered.

c. “I was feeling immense love”

Four participants noted experiencing feelings of love during the dosing sessions. This was articulated in various ways and directed towards different entities depending on the individual.

Most participants classified their feelings as “love” or “immense love”. One participant referred to it through more nuanced terms – “benevolent presence”, where there is a combination of sense of comfort, care and quasi-spiritual presence, which can be broadly understood as an experience of human connection and love.

PSY_HC_13: “*I had a real presence…like a sense of something was next to me… people in the abstract sense… were looking out for me… in a very non-physical way… that was really nice… it… almost felt like, somewhere between abstract and physical because it really felt like there was something there… that was just a very benevolent… presence… I think it’s just human love that I was feeling.”*

Nature was also a salient notion associated with love. Participants reported thoughts and visual imagery involving natural elements, such as ‘thoughts of waves and undulating mountain tops”. These experiences were frequently described as “awe-inspiring” and fostered a sense of “connectedness to [the] natural world”.

d. “An experience that I cannot describe”

Over half of the participants noted a sense of ineffability, in which they were unable to articulate their experiences or find words to adequately convey them. Some participants also acknowledged struggles in unpacking their emotions and making sense of the experience.

PSY_HC_09: *“[The experience is] not something that I can put into words particularly eloquently.”*

**2. Dynamic and enhanced psychological and sensory experiences**

Participants reported diverse and heightened effects on emotional and sensory domains following psilocybin administration. Psychological benefits obtained from these “journeys” were often perceived as personally meaningful and influential in their lives. Narratives of heightened sensory experiences mainly consisted of changes in vision, hearing and taste. Additionally, most participants also remarked on undesirable physiological effects induced by psilocybin.

a. “Moments of positivity and negativity”

A range of emotions was elicited during the acute effects of psilocybin, which can be broadly categorised as either positive or negative.

Common positive descriptions included feeling “euphoric”, “enlightened” and “safe”. Of these descriptions, a “sense of calm” was most frequently expressed (n = 5), which was often associated with a sense of relaxation and inner peace. This sense of serenity and inner peace was often accompanied by an experience of pure awareness, characterised by the perception that there is “just [their] consciousness and everything”. This phenomenon arguably reflected a sense of internal unity, which may also be considered an aspect of ‘mystical’ experiences (17).

PSY_HC_04: “*I felt like my whole body was calm, there's nothing else… I would have kept on sitting in the room for…more than 5 hours [and] not [move] at all. I felt like there wasn't anybody, just my consciousness and feeling everything. There's nothing outside of me…”*

Despite many accounts of positive emotional experiences, negative emotions were noted as well, including feelings of dysphoria and anxiety. Four participants indicated feeling emotionally overwhelmed and disoriented during the sessions, often attributed to a dramatic increase in the volume and speed of their thoughts. For instance, one participant used an analogy of being on a rocket:

PSY_HC_14: “*I felt like I was on, a rocket… the wind was just rushing by and you don’t know where to look because everything is just travelling so fast and [I] feel like I don’t have, time to look… [things were] whooshing straight through and it’s very quick… it seemed overwhelming.”*

All participants experienced multiple types of emotions throughout the sessions. Most notably, two participants reported oscillating emotional states, cycling between brief moments of sadness and happiness.

Moreover, set and setting, especially music, appeared to play an important role in shaping participants’ psychological experiences, where, for example, high-frequency music would lead to a different emotion. One participant also commented on the ability of music to give them directions during the session, which helped him to shift away from negative emotions.

PSY_HC_14: *“…ambient music… helped me to be able to re-steer… I felt that I had directions… if it wasn’t for that music or any music at all… I could’ve spun out of control… gone off track and possibly panic… so I am quite glad that I had the music there.”*

b. “I got a lot out of it”

Most participants (n = 9) revealed that the dosing sessions offered them psychological benefits, with some of these gains persisting beyond the course of psilocybin. These can be categorised into 1) newfound perspectives, 2) clarity of mind and 3) changes in interpersonal relationships.

Seven participants indicated that psilocybin allowed them to gain new perspectives, mainly around their approach to life, and facilitated greater self-insight.

PSY_HC_04: “*…[this experience] just opened up a whole new [case about] what I think, how we come into the world, how we die and how we live our life it completely, I'd say [it] changed my whole perspective.”*

Three participants reported that psilocybin provided an opportunity for them to let go of material concerns and everyday worries. This was often associated with the occurrence of ‘mystical’ phenomena during the sessions. For one individual, this shift was linked to a greater appreciation for natural beauty following an experience of ego-dissolution and visual imagery of an Ecuadorian cave:

PSY_HC_12: “*If there’s such things that exist, so awe-inspiring, and beautiful and magnificent as these caves in Ecuador that would have such natural beauty… I shouldn’t be worried about these… small insignificant things in my life that aren’t gonna worry me in five months’ time...”*

Clarity of mind appeared to mostly stem from the sense of calm and serenity participants gained throughout the dosing sessions. During this state, participants often described finding the world and “things being simpler”, where they felt devoid of responsibilities.

PSY_HC_03: “*My mind… was quite clear during the peak… I didn't have all of the thoughts… responsibilities and things that I would normally have in a day crowding my mind. I knew they existed, but I just wasn't ever thinking about them*.”

Of these participants, many observed that they were able to take these benefits beyond the course of psilocybin and integrate them into their everyday lives. While most participants discussed these in general terms, a few provided specific examples to illustrate their experiences.

PSY_HC_03: “*…a situation [arose] in my work where I was going to need to have a difficult discussion… and I spent some time in that first session thinking about how to have that conversation… I think that really helped because… [for] those situations [that] I find quite difficult normally… I was able to come up with some thoughts that I then built upon the next day before I went on to have that conversation…”*

Furthermore, seven participants reflected on the perspectives psilocybin brought to them in terms of interpersonal relationships. This fostered greater appreciation, connection and compassion for others.

PSY_HC_03: *“…there were moments during the first and second session where I reflected on relationships and was appreciative of the relationships I had… [it was] an opportunity to remind myself and not take those relationships for granted.”*

One participant was able to integrate the perspectives into his life, which enabled him to improve quality of his relationships with friends.

PSY_HC_14: *“…not only is it those changes in yourself but you [are] noticing there [are] changes in the dynamics with your relationships as well around you.”*

c.“The visuals were so strong”

All participants reported experiencing altered visual phenomena during the acute effects of psilocybin. Across these accounts, seven participants recalled having visual imagery. This ranged from geometrical shapes to landscapes to vivid scenes, which often occurred with eyes closed. While this experience was generally understood by participants as a result of their visual sensation, one individual noted difficulty distinguishing them from imagination.

*PSY_HC_12: “when I closed my eyes… the surface the roof the walls were made out of this cushion-y… mushy brain material … and in the very centre of that setting was…barber chairs… and there was a man, sitting down there… a male, non-descript though, there was no discernible facial features, or expressions or mood or vibe or about it… I could just see them swimming around in the chair… putting his hand up… pointing and touching like there was an array of iron man futuristic see-through tablets just in front of him… almost like he was… choosing memories to recount… that was probably the most… absurd or alien-like… vision… throughout the experience…”*

Enhanced visual experiences were endorsed by all participants, often occurring with eyes open. “Things were more vivid and colourful” and colours also became “more saturated, more bright” – an experience that many participants enjoyed.

PSY_HC_12*: “I started to… have.. some visuals… the way I described it was watching a three-D movie without the glasses on, and you can see the red and the blue lines trailing after all the… moving images…”*

Four participants noticed a dose-dependent effect. With higher doses, visual effects had increased intensity. In one case, the speed at which imagery formed increased as well.

PSY_HC_08: *“…amongst the three sessions… the [intensities] of the patterns are definitely different, [on 15 mg it] is very optimal… [on 20 mg] the pattern was very strong so it came very quickly and [disappeared] very quickly…”*

d. “Enhancement of sounds” and more

Most participants discussed enhancements in other sensory modalities. This included auditory sensitivity (n = 5), where participants remarked on how they “could hear things more”. Dissimilar to hallucinatory experiences, participants explained that they became more aware and could hear ambient sounds more clearly, whereas these noises would have normally been imperceptible in everyday contexts.

PSY_HC_06: *“I could hear [people] talking or laughing… even the trip of water from… the air conditioner, I could hear everything but it felt like it was coming from… nearby… that’s the kinds of the sounds [that are] so faint that I would not even know that they were there, but I could hear them during the dose.”*

Five participants also mentioned heightened tactile and gustatory sensations.

PSY_HC_03: *“When I did have those jelly beans, I really appreciated it… I could really taste them… they tasted different to.. regular jelly beans…”*

Several participants experienced synaesthesia as well, where sensory modalities such as hearing, vision and touch crossed over, especially while they were listening to music.

PSY_HC_10: *“Sound had [an]… added a dimension to it… [like] listening to music in like four-D or five-D and… so it kinda felt like there was a sense of feeling and seeing the music… on… top of listening as well.”*

e.“Wasn’t feeling too well”

Although many participants endorsed pleasant sensory experiences, almost all participants identified undesired physiological effects as well. The most common effects were fatigue and altered muscle sensations. Most participants reported fatigue as a non-specific effect of psilocybin, while others saw it as a consequence of the emotionally and physically intense nature of the experience. In several cases, this negatively impacted participants’ engagement with the movement tasks.

PSY_HC_10: *“…all I wanted to do first time around was sleep…… it wasn’t because the balancing was difficult it was more that I was just too sleepy, and too tired, don’t want to do anything.”*

Several participants talked about altered muscle sensations, which included pain, stiffness and restlessness in the legs. One participant shared that there was an unusual sensation in their legs, which made them feel “funny” and “springy”. These altered sensations also influenced performance during movement-based activities.

PSY_HC_03: *“I found my legs get really stiff, particularly my abductors on the outside… they were getting a bit achy and tight from such a short activity… each time… I would just shake my legs out, ‘cause they were feeling quite stiff and sore. And that instantly wasn't a problem once I'd done that…”*

Other common physiological effects mentioned were nausea, headache and reduced appetite. Notably, four participants compared the psilocybin experience to being “drunk”, referencing both physical and cognitive effects, such as “disinhibition” and slowed mental processing.

PSY_HC_08: *“…the state of description would be, that you got really really drunk.”*

**Qualitative semi-structured interview template**

1. Initial impressions

2. Study procedures

3. Differences in motor experiences at different doses

- Motor agency
- Motor control
- Reaction time

4. Effects on body

5. Effects on mind

6. Five senses

7. Other perceptual – synaesthesia, illusions, heightening

8. Thoughts – form, speech, unusual ideas

9. Feelings

10. Dissociation

11. Personality

- Self
- Interpersonal

12. Personal meaning, significance
