# Supplementary material for: After the Trip: A Qualitative Study of Healthy Individuals’ Motor Experiences Following Different Doses of Psilocybin
Source: Brain Behav. 2026 Oct 16;16(10):e71807. doi: 10.1002/brb3.71807 (PMC13674734; doi:10.1002/brb3.71807)
Supplement: Supplementary file 3 [file BRB3-16-e71807-s003.docx]

**Supplementary material**

**Qualitative semi-structured interview template**

1. Initial impressions

2. Study procedures

3. Differences in motor experiences at different doses

- Motor agency
- Motor control
- Reaction time

4. Effects on body

5. Effects on mind

6. Five senses

7. Other perceptual – synaesthesia, illusions, heightening

8. Thoughts – form, speech, unusual ideas

9. Feelings

10. Dissociation

11. Personality

- Self
- Interpersonal

12. Personal meaning, significance
